# Effects of a Reclining Position on Postoperative Dysphagia After Esophagectomy for Esophageal Cancer

**DOI:** 10.3390/jcm14207401

**Published:** 2025-10-20

**Authors:** Takahiro Ariga, Tetsuyuki Nagafusa, Kouji Watanabe, Mami Takahashi, Shunji Takashima, Makoto Hasui, Junko Honke, Sanshiro Kawata, Tomohiro Murakami, Eisuke Booka, Tomohiro Matsumoto, Hirotoshi Kikuchi, Hiroya Takeuchi, Katsuya Yamauchi, Yoshihiro Hiramatsu

**Affiliations:** 1 Department of Rehabilitation Medicine, Hamamatsu University School of Medicine, Hamamatsu 431-3192, Japan; t.ariga@sis.seirei.or.jp (T.A.);; 2 Department of Rehabilitation Medicine, Seirei Mikatahara General Hospital, Hamamatsu 433-8558, Japan; 3 Department of Rehabilitation Medicine, Chutoen General Medical Center, Kakegawa 436-0040, Japan; 4 Department of Rehabilitation Medicine, JA Shizuoka Koseiren Enshu Hospital, Hamamatsu 430-0929, Japan; 5 Department of Perioperative Functioning Care and Support, Hamamatsu University School of Medicine, Hamamatsu 433-3192, Japanhiramatu@hama-med.ac.jp (Y.H.); 6 Department of Surgery, Hamamatsu University School of Medicine, Hamamatsu 433-3192, Japan

**Keywords:** esophagectomy, postoperative pulmonary complications, aspiration

## Abstract

**Background/Objective:** Postoperative dysphagia is a common cause of postoperative pulmonary complications (PPCs) following esophagectomy for esophageal cancer. Although the reclining posture is effective for general dysphagia, its effectiveness after esophagectomy remains unclear. Therefore, we aimed to explore effective approaches in the management of dysphagia after esophagectomy. **Methods:** This study included patients who underwent esophagectomy at the Department of Surgery, Hamamatsu University School of Medicine Hospital between January 2018 and March 2021. For the postoperative swallowing evaluation, the patients underwent a videofluoroscopic swallowing study by drinking 30 mL of liquid in two postures, a 45-degree reclining position (45°R) and a 90-degree upright position (90°U), and assessments were performed using the Penetration–Aspiration Scale. **Results:** Eighty-seven patients participated in the study. Laryngeal penetration and aspiration were, respectively, observed in 19 (21.8%) and 21 patients (24.1%) at 90°U, and in 14 (16.1%) and five patients (5.7%) at 45°R (*p* < 0.05). PPCs occurred in 10 patients (11.5%), and vocal cord paralysis occurred in 22 patients (25.3%). However, after adjusting the feeding conditions based on the results of the swallowing assessment, PPCs after meal initiation occurred in only five of these 10 patients (5.7%). **Conclusions:** Although dysphagia after esophagectomy is observed in approximately half of the patients, including those with minor dysphagia, the risk of aspiration can be reduced by changing the patient’s posture during food consumption. Thus, postural changes may be a useful approach for reducing the risk of PPCs.

## 1. Introduction

Esophageal cancer is the seventh most common cancer worldwide and the eighth most common cause of cancer-related death [1]. Although the efficacy of chemotherapy and chemoradiotherapy for esophageal cancer has been reported, esophagectomy remains the mainstay treatment option for esophageal cancer [2]. However, despite improvements in surgical techniques and postoperative management, the morbidity and mortality rates after esophagectomy for esophageal cancer remain higher than those associated with other thoracic or gastroenterological surgeries [3], with perioperative mortality rates of 2.0–14.0% [4,5,6,7] and morbidity rates of 22.9–50.9% [6,7,8,9]. In recent years, robotic-assisted minimally invasive esophagectomy (RAMIE) has been developed, and a previous study has reported that RAMIE shows significant improvements in clinical outcomes compared with open surgery and conventional minimally invasive esophagectomy (MIE), including reduced postoperative pain, less intraoperative blood loss, and faster recovery [10]. However, postoperative complications after esophagectomy for esophageal cancer—such as pneumonia, anastomotic leakage, recurrent laryngeal nerve paralysis (RLNP), dysphagia, and anastomotic strictures—still need to be carefully considered.

Most importantly, the presence of postoperative pulmonary complications (PPCs) is associated with the overall survival rate [11] and perioperative survival rate [12,13]. Therefore, prevention of PPCs during the postoperative period is extremely important. Preoperative respiratory rehabilitation has been reported to reduce the incidence of PPCs [14], while preoperative sarcopenia is associated with pneumonia after esophagectomy [15]. Thus, prehabilitation (preoperative rehabilitation) is one of the strategies for preventing PPCs. Another strategy is management of postoperative dysphagia. New-onset dysphagia, which is responsible for PPCs, often occurs following esophagectomy. The incidence of dysphagia development has been reported to be 8.2–33.4% [16,17,18,19], and comprehensive evaluations for dysphagia can reduce the incidence of PPCs [16].

Recent reports have gradually recognized that postoperative dysphagia is a frequent postoperative complication, and aspiration prevention is an important strategy for pneumonia prevention. Dysphagia is assessed by videofluoroscopic swallowing study (VFSS) and fiberoptic endoscopic evaluation of swallowing (FEES) [20], but specific assessment methods are not standardized, and the morbidity rate is not clear. In addition, while interventions by a speech-language pathologist [21] and oral intake in the chin-down maneuver [22] have been reported to be effective in dealing with dysphagia, there is still no consensus on effective intervention methods.

Oral intake in a reclining position is a standard intervention for dysphagia [23], and it has been shown to be effective in stroke patients [24] and in patients after oral tumor surgery [25]. Surgical approaches (open, minimally invasive, or robotic) and reconstruction methods (gastric tube or colon interposition) may cause postoperative swallowing dysfunction in this population. However, its efficacy for postoperative dysphagia in patients after esophagectomy for esophageal cancer remains unclear. Therefore, it is necessary to clarify the clinical effectiveness of the reclining posture in preventing aspiration and establishing safe swallowing conditions after esophagectomy for esophageal cancer.

Accordingly, this study primarily aimed to clarify the effectiveness of the reclining position for postoperative dysphagia after esophagectomy for esophageal cancer, by evaluating the frequency and features of postoperative dysphagia using VFSS.

## 2. Materials and Methods

### 2.1. Ethics Statement

All procedures involving human participants followed the ethical standards of the institutional and/or national research committee and the 1964 Declaration of Helsinki and its later amendments or comparable ethical standards. The study was approved by the Institutional Review Board of Hamamatsu University School of Medicine (IRB approval no. 21-344 and date of approval: 12 April 2022). As this was a retrospective study, individual written informed consent was not obtained. Instead, in accordance with institutional ethical guidelines, an opt-out procedure was implemented. Information regarding the study was publicly disclosed, and participants were given the opportunity to decline participation.

### 2.2. Patients

This retrospective observational study included 87 patients who underwent esophagectomy for esophageal cancer with two- or three-field lymphadenectomy at the Department of Surgery, Hamamatsu University Hospital, between January 2018 and March 2021. Clinical data were collected retrospectively from medical records, including age, sex, histological type of tumor, pathological findings, operative time, extent of thoracic/abdominal manipulation and lymph node dissection, reconstruction route, and postoperative complications. Pathological findings were classified according to the tumor-node-metastasis (TNM) classification system of the International Union Against Cancer (UICC, 8th edition). The inclusion criteria were as follows: (1) availability of complete clinical data, and (2) VFSS data obtained in both the 45°R and 90°U positions. The exclusion criteria were as follows: (1) cases in which two-stage surgery was performed, (2) cases in which VFSS in the 45°R and 90°U positions was not performed on the same day, and (3) cases with incomplete data.

### 2.3. Swallowing Assessment

Seven days after surgery, thoracic CT and esophagography were performed by the surgeon. At this point, the presence or absence of swallowing dysfunction was unknown. To avoid aspiration, barium was injected from the mouth side of the anastomosis by using a tube for evaluation during esophagogastrography. If no anastomotic leakage was found, swallowing assessment was performed by a rehabilitation doctor. Swallowing assessments included laryngoscopy and VFSS. A rehabilitation doctor and speech-language pathologist (SLP) with more than 5 years of experience were present during the examination, and the evaluation was performed. In addition, the examinations were conducted by multiple rehabilitation doctors and SLPs, and different examiners were assigned to each patient. However, the examinations were not performed in a blinded manner. The examiners were provided with the patients’ clinical information, and both the examination and evaluation were performed by the same examiner.

Laryngoscopy was used to evaluate vocal cord paralysis, saliva retention in the laryngeal valley and piriform fossa, and pharyngeal constriction. In cases where RLNP was observed, vocal evaluations for characteristics such as hoarseness were also performed. The examination was based on FEES [26]. The scope was inserted through the nasal cavity in a 45° reclining position, and the movements of the soft palate and vocal cords and saliva retention were checked under visualization. Pharyngeal contraction was confirmed by voluntary swallowing. The equipment used was a nasopharyngeal fiberscope (FNL-10RBS, FNL-10RP3; PENTAX, Neo City Mitaka 13F, 3-35-1 Shimorenjaku, Mitaka City, Tokyo, Japan), LED light source device (S281P; PENTAX, Neo City Mitaka 13F, 3-35-1 Shimorenjaku, Mitaka City, Tokyo, Japan), USB camera (S181P; PENTAX, Neo City Mitaka 13F, 3-35-1 Shimorenjaku, Mitaka City, Tokyo, Japan), and USB camera image-processing software (S181P(SW); PENTAX, Neo City Mitaka 13F, 3-35-1 Shimorenjaku, Mitaka City, Tokyo, Japan).

VFSS was performed after laryngoscopy. All patients were examined in the lateral plane at 45°R and 90°U and in the frontal plane at 90°U. In the lateral view, the preparatory and pharyngeal stages were assessed using 5 mL of extremely thick barium paste (Barytgen HD^®^, Fushimi Pharmaceutical Co., Ltd., Marugame, Kagawa, Japan; diluted with water to 40%, *w*/*v* plus Thicken Up thickening agent) and 5 mL and 30 mL of liquid barium (Barytgen HD^®^ diluted with water to 40%, *w*/*v*) in each position. In the frontal view, the esophageal stage was assessed using 5 mL of extremely thick barium paste. The procedure was performed two or three times. The samples (5 mL) were provided by the examiner with a spoon, and 30 mL of liquid was self-administered by the patient in a cup. In cases showing clear aspiration, a decision was made regarding continuation of the procedure.

The VFSS (CUREVISTA; Hitachi Medical Corp., 4-14-1 Soto-kanda Chiyoda-ku Tokyo, 101-0021, Japan) was recorded in video format and validated retrospectively. The severity of dysphagia was assessed using the Penetration–Aspiration Scale (PAS) [27] (Table 1). The PAS describes the degree to which a substance enters the airway and the response to entry or aspiration. The PAS is an eight-level scale, with scores of 1, 2–5, and 6–8 indicating a normal status, penetration (flow into the airway but not past the vocal cords), and aspiration (flow past the vocal cords and into the trachea), respectively. Penetration and aspiration were scored by PAS when 30 mL of liquid was ingested at 45°R and 90°U, respectively. In the lateral view, the ability to chew, degree of pharyngeal residue, and degree of piriform fossa residue were evaluated. Evaluation of the esophageal stage from the frontal view showed residue reflux in the gastric tube. The possibility of removing the residue with a liquid or jelly was also evaluated.

On the basis of the VFSS results, we determined the viability of oral intake, posture setting, and the food form. Patients who were judged as being able to consume food orally were started on oral intake on the day of the examination or the following day after setting the conditions (angle of reclining posture, food content, and presence of thickening). Feeding instructions by a speech pathologist and dietary monitoring by a nurse were provided when necessary.

Cases in which aspiration was observed during the initial evaluation or deemed necessary by the rehabilitation doctor were re-evaluated on the basis of the progress of the diet and the results of direct training. Reassessment was occasionally performed more than once as needed. Depending on the results of the reevaluation, the form of the food was changed or the thickness was modified. Dietary morphology was assessed using the Functional Oral Intake Scale (FOIS) [28] (Table 2), a seven-point scale for oral food tolerance. The scores ranged from 1 (complete dependence on enteral tube feeding) to 7 (tolerating an oral diet without restriction). No enteral tube was used for patients with a score greater than 3.

### 2.4. Statistical Analysis

The Wilcoxon signed-rank test was used as a non-parametric test. All statistical analyses were performed using Statistical Package for Social Science (SPSS^®^) version 27.0 (IBM, Armonk, NY, USA). The significance level was set at *p* < 0.05.

## 3. Results

### 3.1. Patient Characteristics

Eighty-seven patients were included in the study. Patient characteristics are shown in Table 3. The median age was 68 years (range, 43–85 years), and most patients were male (82.8%). The predominant histological type was squamous cell carcinoma (SCC) (80.5%). Approximately half of the patients (43.7%) received neoadjuvant chemotherapy (NAC). Thoracoscopy and laparoscopy were commonly performed in cases involving thoracic and abdominal surgery, respectively (73.6% and 71.3%, respectively). Three-field dissection was usually performed for lymph node dissection (66.7%). Gastric tube reconstruction was performed mostly via the posterior mediastinal route (89.7%). In terms of postoperative morbidity, 22 patients (25.2%) showed vocal cord paralysis and 10 (11.5%) showed PPCs. Of the 10 patients who presented with PPCs, five (5.7%) showed the onset of PPCs after starting oral intake. No perioperative deaths were recorded.

### 3.2. Swallowing Assessment Results

**Figure 1:**
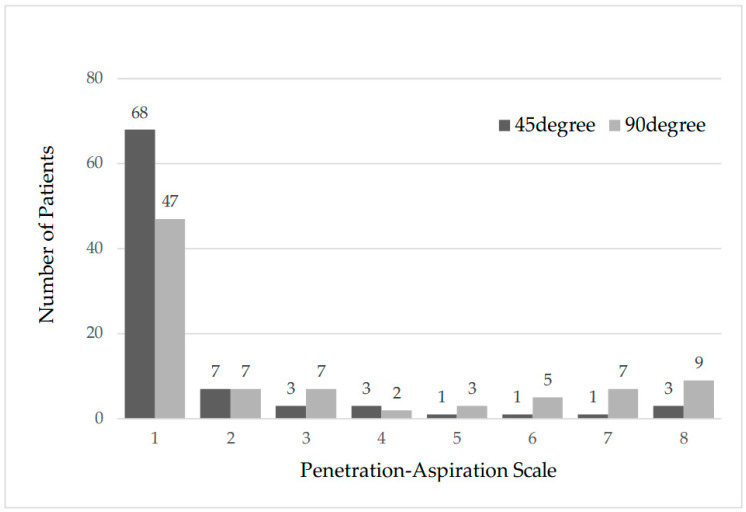
The number of patients with each Penetration–Aspiration Scale (PAS) score in the 45° and 90° swallowing positions.

The median time from surgery to the first VFSS was 9 days (range, 7–30 days). The VFSS results are presented in Table 4 and Figure 1. Forty patients (45.9%) experienced penetration or aspiration (PAS score ≥ 2) when ingesting 30 mL of liquid at 90°U, of which 19 (21.8%) experienced penetration (PAS score 2–5) and 21 (24.1%) experienced aspiration (PAS score 6–8). In contrast, at 45°R, 19 patients (21.8%) experienced penetration and aspiration (PAS score ≥ 2), of which 14 (16.1%) experienced penetration (PAS score 2–5) and 5 (5.7%) showed aspiration (PAS score 6–8). On comparing the two body positions, the PAS score due to postural change was significantly higher at 90°U (*p* < 0.05).

Of the 22 cases with RLNP, 6 (27.3%) and 9 (40.9%) cases showed penetration and aspiration, respectively, at 90°U, while 2 (9.1%) and 2 (9.1%) cases showed penetration and aspiration at 45°R, respectively (Table 4), with significantly more cases of dysphagia at 90°U (*p* < 0.05). One case of aspiration in thick barium paste was observed at 90°U, and none were observed at 45°R. None of the patients had difficulty initiating oral intake because of frequent aspiration or large amounts of barium paste. Based on the VFSS results, 29 patients started eating at 45°R, while others started eating at 90°U. The results for diet are shown in Supplemental Table S1. The median FOIS score at the start of the diet was 4 (range 2–6).

Reevaluation was required in 26 patients (29.9%), and the median interval between the initial evaluation and reevaluation was 10 days (range, 6–43 days). Six patients (6.9%) showed liquid aspiration at 90°U at the time of reevaluation, which was less than the original 21 patients (24.1%). In the reevaluation, 13 of 26 patients showed improvement in comparison with the initial evaluation (Supplemental Table S2). The median FOIS score at discharge was 6 (range 5–7), and all 87 patients were discharged at 90°U and able to continue oral intake (Supplemental Table S1).

As additional analyses, we examined the relationship between aspiration (PAS ≥6) and various clinical variables, including neoadjuvant chemotherapy (NAC), operative approach (thoracotomy/thoracoscopy, laparotomy/laparoscopy), extent of lymphadenectomy, reconstruction route, recurrent laryngeal nerve paralysis (RLNP), and postoperative pulmonary complications (PPCs). No statistically significant associations were observed for most variables; however, RLNP was significantly associated with a higher frequency of aspiration (*p* < 0.05) (Supplemental Table S3).

## 4. Discussion

The present study demonstrated that a 45° reclining posture significantly reduced the risk of aspiration compared with the 90° sitting position in patients after esophagectomy. This finding suggests that a simple positional adjustment during oral intake may contribute to reducing postoperative pulmonary complications, which remain a major cause of morbidity and mortality in this population.

Aspiration after esophagectomy is multifactorial. In our cohort, recurrent laryngeal nerve palsy (RLNP) was strongly associated with increased risk of aspiration, consistent with previous reports that vocal cord immobility contributes to impaired airway protection. Although these were Supplementary Data, the surgical approach (open, minimally invasive, or robotic), reconstruction method, and neoadjuvant therapy did not show significant associations with aspiration, although the sample size was limited. These findings highlight the importance of individualized risk assessment in swallowing rehabilitation.

The incidence of dysphagia in the pharyngeal stage after esophagectomy has been reported to be 8.2–33.4% [16,17,18,19], and its causes include vocal cord paralysis due to RLNP, passage obstruction, or esophageal reflux due to anastomotic stricture. However, dysphagia has also been observed in the population without these factors, suggesting that other mechanisms are involved in the appearance of dysphagia [29]. New postoperative dysphagia mainly appears in the pharyngeal phase rather than the oral phase [30]. Investigations of dysphagia in the pharyngeal region have revealed a reduction in the maximum anterior–posterior diameter of the esophageal inlet and the anterior translation distance of the hyoid bone [31], as well as postoperative changes in the translation distance of the hyoid bone [29]. Dysphagia in the pharyngeal stage accounted for 45.9% of the cases and included mild-to-severe cases. In healthy elderly people without dysphagia, the incidence of penetration and aspiration is 11.4% and 0.6%, respectively [32]. In this study, the incidence of liquid penetration was 21.8% and that of aspiration was 24.1%, suggesting that dysphagia was higher in post-esophagectomy patients than in healthy elderly patients.

Various approaches have been employed for the management of patients with dysphagia, including adjustment of the food textures to be ingested, chin-down maneuvers, and reclining postures. For dysphagia after esophageal cancer treatment, the efficacy of changes in food texture [33] and chin-down maneuver [22] has been reported, but none of the previous studies have evaluated the effects of a reclining posture. Nevertheless, this posture has been reported to be effective in other diseases such as stroke [24] and in patients who have undergone oral tumor surgery [25], and we believe that it can be highly effective for dysphagia after esophageal cancer surgery. The reclining position has been reported to offer the following benefits [25]: (1) Because the trachea is located above the esophagus, it is physically difficult for a bolus to enter the trachea while the patient is asleep. (2) Since the posterior pharyngeal wall is tilted, food slides down the posterior pharyngeal wall and slowly reaches the hypopharynx. (3) Gravity can be used to move food even when active movement is difficult due to tongue movement or congenital disorders. In this study, we assessed the swallowing function under two different body limb positions, 90°U and 45°R, and compared penetration and aspiration of liquids in both positions. The results showed that 45°R significantly reduced penetration and aspiration rates (45.9% vs. 21.8%, *p* < 0.05). Dysphagia was significantly reduced in the reclined position, suggesting its efficacy.

RLNP leads to vocal cord paralysis, which can be a factor in dysphagia [18]. In cases involving RLNP, aspiration was observed in 40.9% of patients at 90°U, and the presence of RLNP was a risk factor for dysphagia. However, of the 24 patients showing aspiration at 90°U, only nine had RLNP, indicating that patients with dysphagia do not necessarily have RLNP. Several mechanisms can explain aspiration in vocal cord paralysis, but the most likely mechanisms are glottal closure and laryngeal sensory disturbances [34]. The glottis cannot be closed; therefore, liquids and boluses are aspirated into the trachea as they enter the larynx. However, in cases with no impairment in laryngeal elevation or swallowing guidance other than vocal cord paralysis, aspiration did not occur because laryngeal entry did not occur.

Ten patients (11.5%) in our study population had PPCs. The incidence of PPCs after surgery for esophageal cancer has been reported to range from 10.9% to 22.5% [35,36], which is consistent with the findings of the present study. However, pneumonia after the start of oral intake was observed in only five patients (5.7%). Comprehensive swallowing interventions, such as oral intake in a reclined position and changes in food form, were performed when necessary, which is thought to have contributed to the results.

Interventions such as swallowing assessment and training by speech-language pathologists have been reported to be effective in reducing the time to initiation of oral intake and the length of hospital stay [21], but bedside swallowing assessment is not sensitive, and aspiration detection requires evaluation by contrast swallowing [18]. In addition to methods for aspiration, VFSS is an excellent approach for studying the dynamics of various physical foods from the oral to the esophageal phase and the effectiveness of positioning, such as cervical rotation and chin-down maneuvers.

In this study, we believed that the use of swallowing contrast studies in all patients allowed for appropriate selection of objective dysphagia, posture, and food form, and that setting safe feeding conditions could reduce the morbidity of pneumonia after the initiation of meals. Based on these findings, our institution has incorporated swallowing contrast studies at both 45° and 90° reclining positions into the postoperative protocol for patients after esophagectomy for esophageal cancer, using these evaluations to determine the timing of oral intake initiation, posture setting, and the appropriate food form. This protocol has successfully contributed to reducing the risk of aspiration pneumonia in these patients.

All 87 patients were able to eat in a 90°U position at the time of discharge, without the need for a 45°R position. Therefore, no specific instruction regarding the reclining position was required for eating at home. Consequently, the effectiveness of the reclining position during meals after discharge remains unclear. In addition, the incidence of pulmonary complications after discharge is also unknown, because no follow-up survey was conducted.

Future research should include prospective, multicenter studies with larger cohorts to validate the efficacy of reclining posture in reducing aspiration. Incorporating preoperative VFSS would help clarify the direct impact of surgery on swallowing function. Longitudinal follow-up studies are needed to evaluate the durability of postural interventions and their effect on long-term nutritional outcomes, quality of life, and pulmonary complications. Additionally, randomized controlled trials comparing reclining posture with other compensatory strategies, such as chin-tuck or head rotation, may help establish evidence-based guidelines for postoperative swallowing rehabilitation.

This study has several limitations. The first limitation is potential selection bias. The selection criteria for cases may have been arbitrary or restrictive, and the study population may have been limited to patients with favorable postoperative courses who were able to undergo swallowing examinations. Furthermore, some cases were excluded because of missing data, which may have introduced additional bias. Because this was a single-center study, institutional treatment policies and the surgeons’ approaches may have influenced patient selection, and the findings may not be generalizable to other institutions or populations. The second limitation is potential observer bias. Multiple examiners and evaluators participated in the swallowing examinations, which might have resulted in inter-rater variability. Moreover, the specialists who performed VFSS were not blinded to the patients’ clinical information, which could have further introduced bias. The third limitation is the relatively small sample size, which limited the statistical power to detect associations between surgical and oncological factors. The fourth limitation is that preoperative swallowing function was not evaluated; therefore, some patients may have had dysphagia before surgery. Consequently, preoperative factors such as sarcopenia due to malnutrition from poor digestion, asymptomatic cerebral infarction, and weight loss or nutritional deficiency caused by adverse effects during neoadjuvant chemotherapy (NAC) may have contributed to dysphagia. In such cases, postoperative dysphagia might not have been solely attributable to surgical intervention. The fifth limitation is that VFSS was performed only in the early postoperative period (on postoperative day 7); therefore, long-term changes in swallowing function and nutritional status were not evaluated.

## 5. Conclusions

Although dysphagia after esophagectomy for esophageal cancer is observed in approximately half of the patients, including even mild cases, the risk of aspiration can be reduced by accommodating oral intake in a reclined position. Oral intake in a 45-degree reclining position is expected to reduce the incidence of post-meal PPC after meal initiation.

## Figures and Tables

**Table 1:** Penetration–Aspiration Scale (PAS).

Category	Score	Descriptions
Normal	1	Contrast does not enter the airway
Penetration	2	Contrast enters the airway, remains above vocal folds; no residue
	3	Contrast remains above the vocal folds; visible residue present
	4	Contrast contacts vocal folds; no residue
	5	Contrast contacts vocal folds; visible residue present
Aspiration	6	Contrast passes glottis; no subglottic residue visible
	7	Contrast passes glottis; visible subglottic residue despite patient’s response
	8	Contrast passes glottis; visible subglottic residue; no patient response

**Table 2:** Functional Oral Intake Scale (FOIS).

Score	Performance	Implication	Deficit
1	Aspirates saliva/tube-dependent	Nothing by mouth	Profound
2	Tube-dependent	Nothing by mouth/minimal trials	Profound
3	Tube-dependent	Full trials by mouth	Severe
4	Total oral	Single-texture trials	Moderate
5	Total oral	Multiple-texture trials	Mild
6	Total oral	By mouth/restrictions	Minimal
7	Regular diet	By mouth/no restrictions	None

**Table 3:** Sociodemographic and clinical characteristics.

	n	%
*Sociodemographic*		
Age (years), median (range)	68 (43–85)	
Sex		
Male	72	82.8
Female	15	17.2
Interval between surgery and VFSS (days), median (range)	9 (7–30)	
*Clinical characteristics*		
Histological type		
Squamous cell carcinoma	70	80.5
Adenocarcinoma	17	19.5
Pathological tumor stage (UICC 8th)		
T0	4	4.5
T1a	15	11.5
T1b	24	27.6
T2	9	10.3
T3	31	34.5
T4a	4	4.6
T4b	0	0
Pathological nodal stage (UICC 8th)		
N0	33	37.9
N1	28	32.2
N2	10	11.5
N3	16	18.3
Location of tumor		
Cervical	2	2.3
Upper thoracic	13	14.9
Middle thoracic	28	32.2
Lower thoracic	28	32.2
Abdominal	16	18.4
*Treatment*		
Neoadjuvant chemotherapy		
Yes	38	43.7
No	49	56.3
Operation time (min), median (range)	542 (402–748)	
Operative approach		
Thoracotomy	23	26.4
Thoracoscopy	64	73.6
Laparotomy	25	28.7
Laparoscopy	62	71.3
Abdominal/transhiatal	0	0.0
Lymphadenectomy		
3-Field	58	66.7
2-Field	29	33.3
Reconstruction		
Posterior mediastinal	78	89.7
Antethoracic	0	0.0
Retrosternal	9	10.3
*Complications in postoperative period*		
Vocal cord palsy		
Bilateral	0	0.0
Left	17	19.5
Right	5	5.7
Postoperative pulmonary complication		
Yes	10	11.5
No	77	88.5

VFSS, videofluoroscopic swallowing study.

**Table 4:** PAS scores for the 45-degree reclining position and 90-degree upright position.

		90-Degree		45-Degree		*p* Value ^a^
		n	%	n	%	
PAS All (n = 88)						*p* < 0.05
1	Normal	47	54.0	68	79.2	
2–5	Penetration	19	21.8	14	16.1	
6–8	Aspiration	21	24.1	5	5.7	
RLNP (n = 22)						*p* < 0.05
1	Normal	7	31.8	18	81.8	
2–5	Penetration	6	27.3	2	9.1	
6–8	Aspiration	9	40.9	2	9.1	

PAS, Penetration-Aspiration Scale; RLNP, recurrent laryngeal nerve paralysis. ^a^ Wilcoxon signed-rank test.

## Data Availability

The original contributions presented in this study are included in the article/Supplementary Materials. Further inquiries can be directed to the corresponding author.

## Supplementary Materials

The following supporting information can be downloaded at https://www.mdpi.com/article/10.3390/jcm14207401/s1, Table S1. Diet after surgery; Table S2. PAS score on re-evaluation; Table S3. Association between clinical factors and aspiration on VFSS (PAS ≥ 6) at 90°U.
