# Supplementary material for: Effects of a Reclining Position on Postoperative Dysphagia After Esophagectomy for Esophageal Cancer
Source: J Clin Med. 2025 Oct 20;14(20):7401. doi: 10.3390/jcm14207401 (PMC12565057; doi:10.3390/jcm14207401)
Supplement: Supplementary file 1 [file jcm-14-07401-s001.zip › supplmentary 1-2.pdf]

Supplemental Table S1. Diet after surgery.

	n	%
Interval between surgery and intake (days), median (range)	10 (8-36)	
FOIS score, median (range)		
First intake after surgery	4 (2-6)	
Discharge	6 (5-7)	
Reclining position		
45°R	29	*33.3*
90°U	58	*66.7*
Speech-language pathologist intervention		
Yes	55	*63.2*
No	32	*36.8*

FOIS, functional oral intake scale; 45°R, 45-degree reclining position; 90°U, 90-degree upright position.

Supplemental Table S2. PAS score on re-evaluation

		1st VFSS		2nd VFSS	
		n	%	n	%
PAS					
Re-evaluation (n = 26)					
1	Normal	3	*11.5*	7	*26.9*
2-5	Penetration	4	15.4	12	46.2
6-8	Aspiration	19	*73.1*	7	26.9

PAS, Penetration–Aspiration Scale; VFSS, videofluoroscopic swallowing study
