# Supplementary material for: Effects of a Reclining Position on Postoperative Dysphagia After Esophagectomy for Esophageal Cancer
Source: J Clin Med. 2025 Oct 20;14(20):7401. doi: 10.3390/jcm14207401 (PMC12565057; doi:10.3390/jcm14207401)
Supplement: Supplementary file 1 [file jcm-14-07401-s001.zip › jcm-3897636-supplementary.pdf]

Supplementary Table S3. Association between clinical factors and aspiration on VFSS (PAS ≥6) at 90°U

Factor	n	Aspiration (+)	Aspiration (−)	p-value[a]
NAC (yes/no)	38/49	15/25	23/24	0.196
Operative approach (Thoracotomy/Thoracoscopy)	23/64	12/28	11/36	0.325
Operative approach (Laparotomy/Laparoscopy)	25/62	12/28	13/34	0.498
Lymphadenectomy(3-field/2-field)	57/30	27/13	30/17	0.448
Reconstruction (posterior mediastinal/ retrosternal)	78/9	35/5	43/4	0.397
RLNP (yes/no)	22/65	15/25	7/40	0.015
PPCs (yes/no)	10/77	7/33	3/44	0.100

NAC.Neo-Adjuvant Chemothrapy;PAS,Penetration-Aspiration Scale; RLNP,Recurrent Laryngeal Nerve Paralysis;PPCs,Postoperative Pulmonary Complications;

a Wilcoxon signed-rank test
